# Overview of Cancer Rehabilitation Provided in Palliative Care Units in Japan: A Nationwide Survey

**DOI:** 10.1298/ptr.25-E10376

**Published:** 2026-06-04

**Authors:** Takuya FUKUSHIMA, Tetsuya TSUJI, Kazunori TAKASHIMA, Jiro NAKANO

**Affiliations:** 1 Faculty of Rehabilitation, Kansai Medical University, Japan; 2 Department of Rehabilitation Medicine, Keio University School of Medicine, Japan; 3 Department of Rehabilitation, Faculty of Health Sciences, Hiroshima Cosmopolitan University, Japan

**Keywords:** Cancer rehabilitation, Palliative care unit, Advanced cancer, Intervention

## Abstract

**Objectives:**

We aimed to clarify the current state of cancer rehabilitation in palliative care units (PCUs) in Japan, thus helping to improve cancer rehabilitation and effectively address patients’ complex needs.

**Methods:**

A nationwide questionnaire survey was conducted, targeting 462 hospitals in Japan that met the eligibility criteria for reimbursement of inpatient fees for PCUs and had submitted notifications to local health authorities. The survey focused on cancer types, intervention types, duration and frequency of interventions, and other characteristics of cancer rehabilitation in PCUs.

**Results:**

Responses were received from 265 facilities (response rate, 57.4%), of which 242 (91.3%) provided cancer rehabilitation in their PCU. Cancer rehabilitation in Japanese PCUs primarily targeted patients with lung, hepatobiliary and pancreatic, colorectal, gastric, and breast cancers. Interventions included relaxation therapy, walking, basic mobility training, resistance exercises, and range-of-motion exercises. Support for quality of life, *ikigai* (a Japanese concept for a sense of meaning and purpose in life), and temporary discharge from the PCU were identified as distinctive elements of rehabilitation in palliative care. In many facilities, sessions lasted 20–40 min, with interventions provided 3–4 times per week.

**Conclusions:**

The findings highlight the need for further evaluation of the effectiveness of interventions tailored to unmet medical needs, as well as the development of evidence stratified by cancer type and disease stage. Such evidence may inform future policies to strengthen rehabilitation within PCUs.

## Introduction

Supportive care, including cancer rehabilitation, is crucial for preserving patients’ dignity during the palliative phase^1)^. During this stage, patients and their caregivers often encounter numerous unmet needs, such as deteriorating physical function, emotional distress, and difficulties with daily activities, all of which can significantly affect their quality of life (QoL)^2–4)^. Previous studies have shown that integrating cancer rehabilitation into palliative care can improve QoL and physical function, alleviate symptom burden, and provide support to both patients and caregivers during this challenging phase^1,5)^. However, the core challenge lies in developing and implementing a cancer rehabilitation system that effectively addresses these complex needs.

Recent reviews and multinational studies consistently report the growing recognition of palliative cancer rehabilitation as a vital component of comprehensive cancer care^1,6)^. However, rehabilitation services remain underused, especially among socially vulnerable populations, owing to funding restrictions, lack of guidelines, and insufficient professional training^7–10)^. A study examining the current state of cancer rehabilitation in palliative care units (PCUs) in Japan revealed that, while cancer rehabilitation is implemented in approximately 90% of facilities, nearly 65% of these facilities acknowledge its inadequacy^11)^. Factors such as ineligibility for medical fee reimbursement for disease-specific rehabilitation and a shortage of rehabilitation staff may contribute to this inadequate utilization^11)^. Therefore, a comprehensive understanding of the current state of cancer rehabilitation in PCUs is crucial to improve care.

In other clinical settings, including hospitals and outpatient settings, the scope and content of rehabilitation vary depending on the treatment phase^12,13)^. Accordingly, rehabilitation in PCUs may differ significantly from that in these settings. However, to the best of our knowledge, detailed assessments of cancer rehabilitation practices within PCUs, such as targeted cancer types, specifics of intervention content, intervention frequencies, and session durations, are limited. Establishing foundational data on cancer rehabilitation in PCUs is an urgent issue and will be crucial to guide future developments.

Hence, in this study, we aimed to examine the current practices and characteristics of cancer rehabilitation in PCUs in Japan through a nationwide survey. This study was based on the same nationwide survey dataset as that in a previous report^11)^. However, while the previous study examined the prevalence of cancer rehabilitation implementation and perceived barriers among hospitals with PCUs, this present study focused exclusively on facilities providing cancer rehabilitation in PCUs and explored the characteristics of actual rehabilitation practices. Evidence from this study is expected to inform policy changes aimed at improving cancer rehabilitation, including the development of appropriate reimbursement systems, additional service fees, and the integration of rehabilitation staff into PCUs.

## Methods

### Study design and population

We conducted a nationwide questionnaire survey targeting 462 hospitals in Japan that met the facility standards set by the Ministry of Health, Labour and Welfare. Eligible hospitals were also those that were formally authorized as PCUs under the national reimbursement system and that had officially submitted notifications for the PCU inpatient fee. Hospitals that did not submit these notifications, such as those providing palliative care in non-designated wards or through palliative care teams without PCU designation, were excluded because they are not recognized as PCUs for inpatient fee reimbursement. The survey was distributed to the heads of rehabilitation departments and included professionals such as physical therapists (PTs), occupational therapists (OTs), speech and language therapists (SLTs), and physiatrists.

This study received ethical approval from the Kansai Medical University (approval number: 2023418). All procedures adhered to the ethical principles outlined in the national regulations and the Declaration of Helsinki (1964) and its subsequent revisions. Completion of the questionnaire was considered informed consent.

### Survey method and questionnaire

A multidisciplinary team of physiatrists, PTs, and OTs specializing in cancer rehabilitation developed the survey questionnaire. The PTs initially drafted the questionnaire items based on their clinical experience and relevance to palliative care rehabilitation. Subsequently, these draft items were refined through iterative discussions among physiatrists, PTs, and OTs. The final items were determined once consensus was reached among all members of the study team (Supplementary Material 1). In June 2024, the survey questionnaire was distributed by postal mail to each eligible hospital, with a request for completion by a rehabilitation professional in a managerial or supervisory position. Hospitals that did not respond within 3 weeks of the initial mailing were sent a reminder.

The survey collected comprehensive data on facility characteristics, including the location of facilities (Hokkaido, Tohoku, Kanto, Chubu, Kansai, Chugoku, Shikoku, Kyushu, and Okinawa), hospital type (university hospital, cancer center, general hospital, or other), total bed capacity (≤300, 301–600, 601–1000, or ≥1001 beds), PCU bed capacity (0, 1–10, 11–30, 31–60, or ≥61 beds), rehabilitation staff (presence of physiatrists, the number of PTs, OTs, and SLTs), and therapists who completed the Cancer Rehabilitation Educational Program for Rehabilitation (CAREER). To account for facilities that may have been in a transitional phase at the time of the survey (e.g., temporary closure or transition of PCU beds), “0 PCU beds” was included as a response option. CAREER is a specialized training program in Japan designed to prepare rehabilitation professionals to provide cancer-specific care^14,15)^. The workshop requires participation of multiple staff members per institution, including 1 medical doctor, 1 nurse, and therapists. The workshop includes an e-learning lecture, followed by a 1-day case review and group work. The lectures provide an overview of the evidence and practice of cancer rehabilitation, including palliative care. During the case review and group work, participants discuss the management and treatment of cancer-related conditions in small groups^16)^.

As part of the detailed survey, we investigated the types of professionals involved in rehabilitation at PCUs (PTs, OTs, or SLTs), implementation system (exclusively assigned to PCU, working in concurrent roles, or both), cancer type, intervention type, daily number of patients receiving PT, OT, or SLT (0, 1–5, 6–10, 11–15, 16–20, or ≥21), intervention time (<20, 20–40, or >40 min), and intervention frequency (1–2, 3–4, 5–6, or 7 times per week).

### Statistical analysis

Data were summarized using median values with interquartile ranges for continuous variables and counts with percentages for categorical variables. To assess potential nonresponse bias, the background characteristics (location of facilities, hospital type, and number of beds) of hospitals with and without survey responses were compared using the chi-squared test. Facilities that reported having no PCU beds were excluded from analyses requiring PCU bed capacity. A descriptive analysis was conducted to examine various aspects of cancer rehabilitation within PCUs, including the types of professionals involved in rehabilitation, implementation systems, cancer types, intervention types, intervention durations, intervention frequencies, and hospital characteristics. For analyses describing the characteristics of responding facilities and the time and frequency of interventions, percentages were calculated using the total number of responding facilities as the denominator. The proportion of “no answer” responses was also calculated. Similarly, for analyses involving items that allowed multiple responses, such as cancer type and type of interventions, percentages were calculated using the total number of responding facilities as the denominator. Facilities were stratified by the type of facilities, number of PCU beds, presence of physiatrists (yes or no), and rehabilitation implementation system. We examined associations between these facility characteristics and cancer types, intervention types, intervention frequencies, and intervention durations using chi-squared tests. To account for multiple comparisons, the Bonferroni correction was applied, and adjusted P-values were reported. Statistical significance was defined as P <0.05. All statistical analyses were performed using IBM SPSS Statistics version 27 (IBM, Armonk, NY, USA).

## Results

Of the 462 hospitals invited to participate in the survey, 265 institutions returned completed questionnaires, resulting in a 57.4% response rate. Comparisons of background characteristics between responding and nonresponding facilities are presented in Table 1. No significant differences were observed in the location of facilities, hospital type, or number of beds. Of these responses, 190 were obtained through initial mailing, and 75 were obtained through follow-up mailing. Among the responding facilities, 242 (91.3%) provided cancer rehabilitation within their PCUs.

**Table 1.** Comparison of the background characteristics of responder and nonresponder facilities

	Nonresponder (n = 197)	Responder (n = 265)	P-value
Location of facilities			0.60
Hokkaido	8 (4.1%)	16 (6.0%)	
Tohoku	10 (5.1%)	20 (7.5%)	
Kanto	54 (27.4%)	62 (23.4%)	
Chubu	30 (15.2%)	35 (13.2%)	
Kansai	34 (17.3%)	51 (19.2%)	
Chugoku	13 (6.6%)	22 (8.3%)	
Shikoku	6 (3.0%)	13 (4.9%)	
Kyushu and Okinawa	42 (21.3%)	46 (17.4%)	
Type of facilities			0.22
University hospital	6 (3.0%)	6 (2.3%)	
Cancer center	5 (2.5%)	10 (3.8%)	
General hospital	179 (90.9%)	241 (90.9%)	
Other	7 (3.6%)	4 (1.5%)	
No answer	0 (0.0%)	4 (1.5%)	
Number of total beds			0.35
<300 beds	98 (49.7%)	117 (44.2%)	
301–600 beds	73 (37.1%)	117 (44.2%)	
601–1000 beds	24 (12.2%)	26 (9.8%)	
>1001 beds	2 (1.0%)	5 (1.9%)	
Number of PCU beds			0.45
0 bed	2 (1.0%)	2 (0.8%)	
1–10 beds	12 (6.1%)	12 (4.5%)	
11–30 beds	167 (84.8%)	222 (83.8%)	
31–60 beds	14 (7.1%)	18 (6.8%)	
>61 beds	0 (0.0%)	1 (0.4%)	
No answer	2 (1.0%)	10 (3.8%)	

Data are presented as n (%). The denominator used to calculate percentages was the total number of eligible facilities in each group. All variables were based on single-response items.

PCU, palliative care units

In terms of institutional characteristics, general hospitals accounted for the majority of responses (218, 90.1%), and most facilities reported having 11–30 PCU beds (206, 85.1%). Physiatrists were present in 90 (37.2%) facilities. Both the total number of rehabilitation staff and the number of those who had completed CAREER were highest among PTs, followed by OTs and SLTs. Within PCUs, PTs (231, 95.5%) were the most common rehabilitation staff, followed by OTs (194, 80.2%) and SLTs (164, 67.8%).

In terms of cancer rehabilitation systems in PCUs, working in concurrent roles was the most common implementation system (216, 89.3%) (Table 2).

**Table 2.** Characteristics of facilities and rehabilitation staff providing cancer rehabilitation in PCU

Type of facilities	
University hospital	6 (2.5%)
Cancer center	10 (4.1%)
General hospital	218 (90.1%)
Other	4 (1.7%)
No answer	4 (1.7%)
Number of total beds	
<300 beds	112 (46.3%)
301–600 beds	105 (43.4%)
601–1000 beds	21 (8.7%)
>1001 beds	4 (1.7%)
Number of PCU beds	
0 bed	0 (0.0%)
1–10 beds	10 (4.1%)
11–30 beds	206 (85.1%)
31–60 beds	18 (7.4%)
>61 beds	1 (0.4%)
No answer	7 (2.9%)
Existence of physiatrists	
Yes	90 (37.2%)
No	149 (61.6%)
No answer	3 (1.2%)
Number of rehabilitation staffs	
PT	16 [10–28]
OT	7 [4–14]
SLT	4 [2–6]
Number of rehabilitation staffs who had completed the CAREER program	
PT	6 [2–11]
OT	2 [1–5]
SLT	1 [0–3]
Types of rehabilitation staffs involved in PCU (multiple answers allowed)	
PT	231 (95.5%)
OT	194 (80.2%)
SLT	164 (67.8%)
Cancer rehabilitation systems in PCU	
Exclusively assigned to PCU	6 (2.5%)
Working in concurrent roles	216 (89.3%)
Both	20 (8.3%)

Data are presented as n (%) or median [interquartile range]. Percentages were calculated using n = 242 facilities that provide cancer rehabilitation in PCU as the denominator. “No answer” responses are shown separately, and their proportions were calculated as well. All variables were based on single-response items.

PCU, palliative care units; PT, physical therapists; OT, occupational therapists; SLT, speech and language therapists; CAREER, Cancer Rehabilitation Educational program for Rehabilitation teams

### Cancer types and rehabilitation interventions in the PCU

Among patients receiving cancer rehabilitation in PCUs, lung cancer was the most common cancer type (204, 84.3%), followed by hepatobiliary and pancreatic cancers (190, 78.5%), colorectal cancer (172, 71.1%), gastric cancer (161, 66.5%), and breast cancer (101, 41.7%) (Fig. 1).

**Fig. 1.**
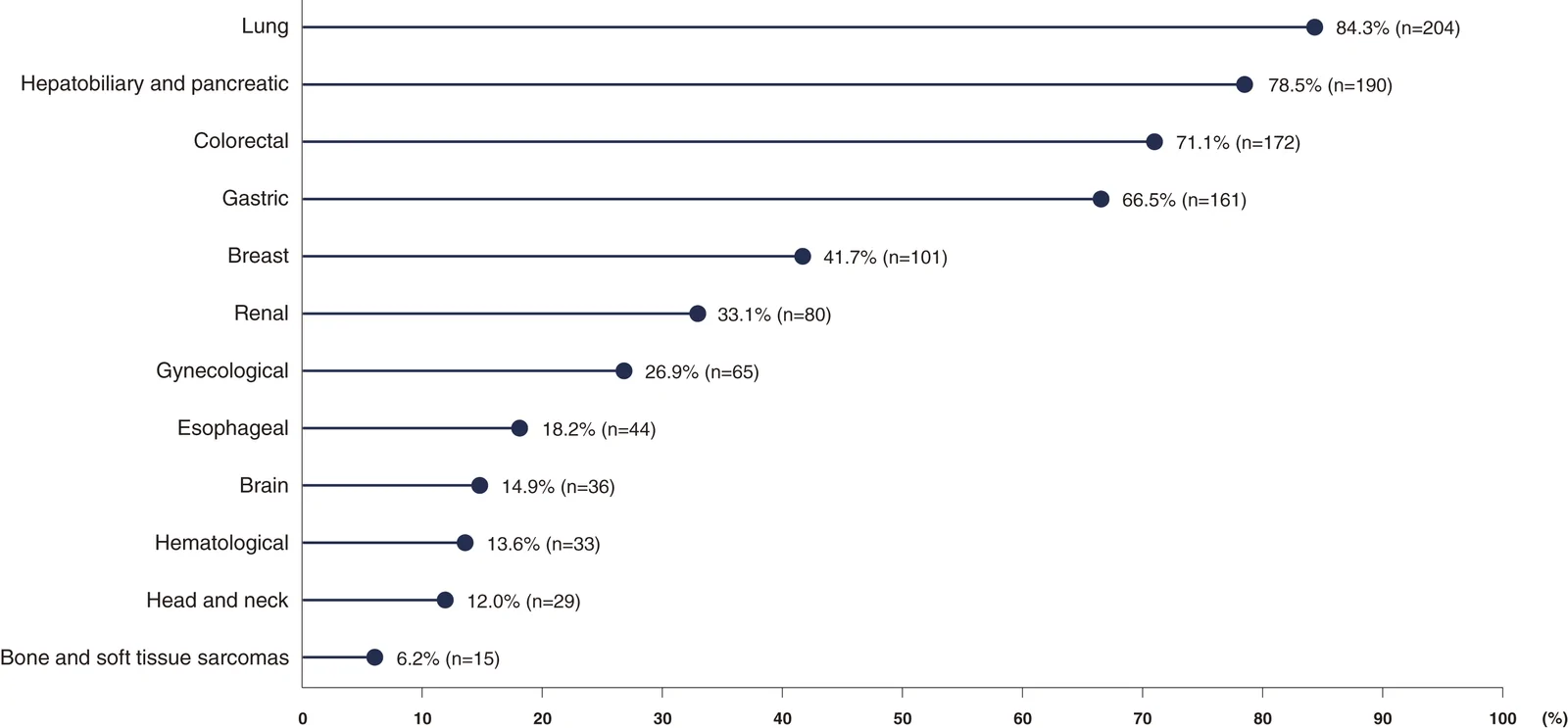
Types of cancer for which cancer rehabilitation is provided in PCUs. The top 5 cancer types in PCUs at each facility are shown as percentages calculated using the 242 facilities that reported providing cancer rehabilitation as the denominator. Multiple responses were allowed (up to 5). PCU, palliative care unit

Regarding the types of interventions, relaxation therapy was the most common (169, 69.8%), followed by walking exercises (159, 65.7%), basic mobility training (134, 55.4%), resistance exercises (128, 52.9%), and range-of-motion (ROM) exercises (122, 50.4%). In addition, support for QoL and *ikigai* (a Japanese concept for a sense of meaning and purpose in life) was provided in 86 (35.5%) facilities. Support for returning home and temporary discharge from the PCU were also included (87, 36.0%) (Fig. 2). Most associations between facility characteristics and cancer types or rehabilitation interventions were not significant. However, a significant association was observed between the number of beds in the PCU and the use of relaxation therapy. Additionally, the rehabilitation implementation system was significantly associated with the use of basic mobility training and support for QoL and *ikigai* (Supplementary Material 2).

**Fig. 2.**
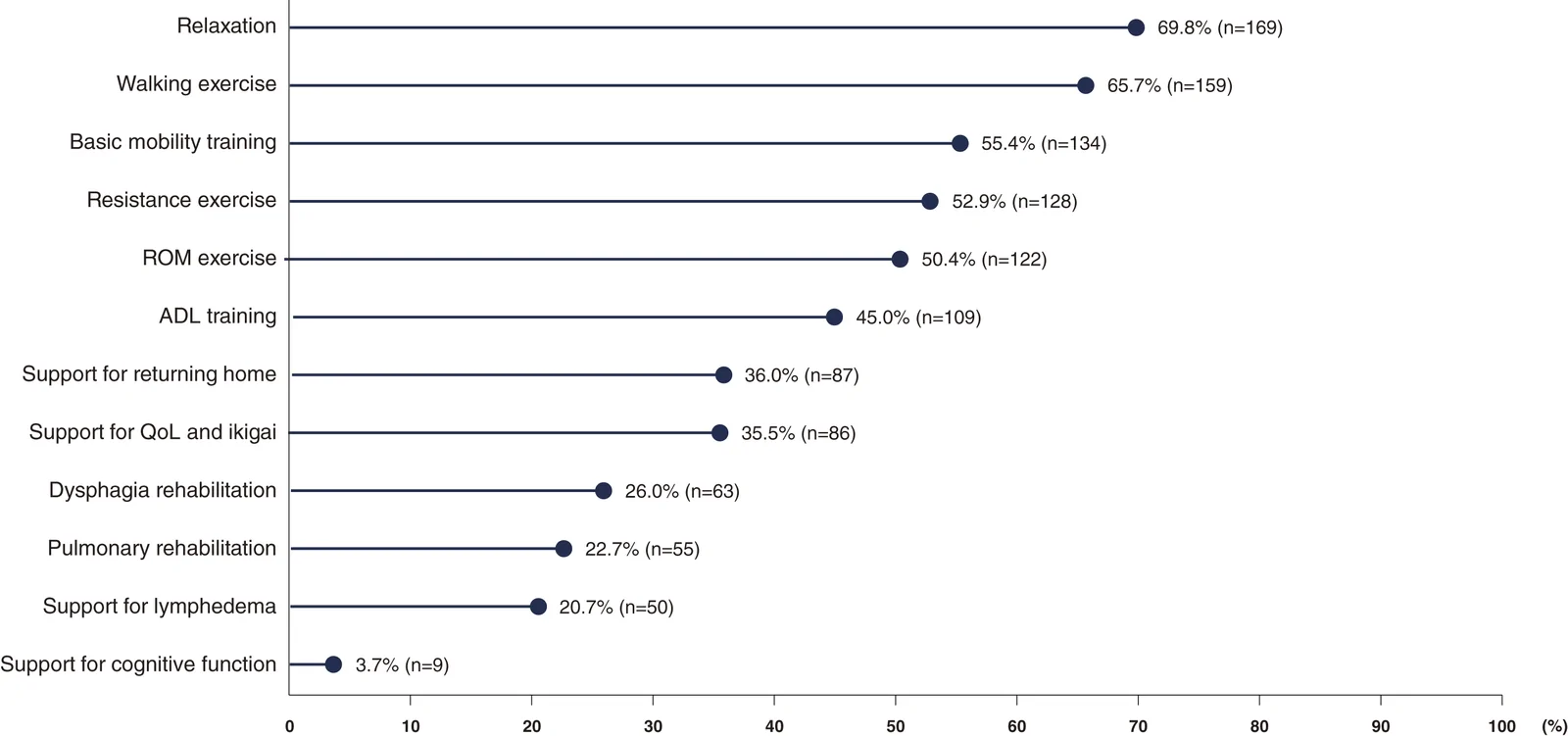
Types of intervention for which cancer rehabilitation is provided in PCUs. The top 5 intervention types in PCUs at each facility are shown as percentages calculated using the 242 facilities that reported providing cancer rehabilitation as the denominator. Support for QoL and *ikigai* refers to rehabilitation-related support aimed at maintaining or enhancing QoL and *ikigai* (a Japanese concept equivalent to meaning and purpose in life). This support is provided in an individualized and patient-centered manner, as defined by each facility. Multiple responses were allowed (up to 5). ROM, range of motion; ADL, activities of daily living; QoL, quality of life; PCU, palliative care unit

### Number of patients, time of intervention, and frequency of cancer rehabilitation in PCUs

The most frequently occurring number of patients receiving PT per day was 1–5 (144, 59.5%), followed by 6–10 (51, 21.1%) and 0 (23, 9.5%). For OT, 1–5 patients per day was most common (117, 48.3%), followed by 0 (77, 31.8%) and 6–10 (20, 8.3%). For SLT, the most frequent number of patients per day was 0 (131, 54.1%), followed by 1–5 (80, 33.1%) (Table 3). Regarding intervention time, most facilities performed the intervention within 20–40 min (174, 71.9%), followed by <20 min (57, 23.6%) and >40 min (8, 3.3%) (Table 3). For intervention frequency, 3–4 times per week was the most common (119, 49.2%), followed by 5–6 times per week (64, 26.4%), 1–2 times per week (51, 21.1%), and daily (6, 2.5%) (Table 3). The facility characteristics were not significantly associated with the time and frequency of interventions (Supplementary Material 2).

**Table 3.** Number of patients, intervention time, and frequency of cancer rehabilitation in the PCU

Daily number of patients receiving PT	
0	23 (9.5%)
1–5	144 (59.5%)
6–10	51 (21.1%)
11–15	13 (5.4%)
16–20	1 (0.4%)
≥21	1 (0.4%)
No answer	9 (3.7%)
Daily number of patients receiving OT	
0	77 (31.8%)
1–5	117 (48.3%)
6–10	20 (8.3%)
11–15	4 (1.7%)
16–20	1 (0.4%)
≥21	1 (0.4%)
No answer	22 (9.1%)
Daily number of patients receiving SLT	
0	131 (54.1%)
1–5	80 (33.1%)
6–10	3 (1.2%)
11–15	1 (0.4%)
16–20	1 (0.4%)
≥21	0 (0.0%)
No answer	26 (10.7%)
Intervention time, per session	
<20 min	57 (23.6%)
20–40 min	174 (71.9%)
>40 min	8 (3.3%)
Others	1 (0.4%)
No answer	2 (0.8%)
Intervention frequency	
1–2 times/week	51 (21.1%)
3–4 times/week	119 (49.2%)
5–6 times/week	64 (26.4%)
7 times/week	6 (2.5%)
No answer	2 (0.8%)

Data are presented as n (%). Percentages were calculated using n = 242 facilities that provide cancer rehabilitation in PCU as the denominator. “No answer” responses are shown separately, and their proportions were calculated as well. All variables were based on single-response items.

PCU, palliative care units; PT, physical therapists; OT, occupational therapists; SLT, speech and language therapists

## Discussion

Although cancer rehabilitation is implemented across PCUs in Japan, the current situation is still considered inadequate^11)^. The findings of this study suggest that the number of patients receiving rehabilitation is relatively low compared to the number of PCU beds. It is crucial to understand the current state of cancer rehabilitation in PCUs to improve care. However, the details of how cancer rehabilitation is implemented in PCUs remain unclear. To our knowledge, this is the first study to collect foundational nationwide data on this topic, with the aim of informing future development of cancer rehabilitation in PCUs.

In Japanese PCUs, lung cancer emerged as the most common target cancer type, followed by hepatobiliary and pancreatic cancers, colorectal cancer, gastric cancer, and breast cancer. This distribution is similar to that seen in inpatient settings in Japan, where lung, colorectal, gastric, and hepatobiliary and pancreatic cancers are among the 5 most frequently treated cancer types^12)^. This pattern is also consistent with cancer epidemiology in Japan, where colorectal, lung, gastric, and breast cancers are among the most frequently diagnosed cancers^17)^. A similar trend is observed internationally; for example, in South Korea’s PCUs, lung cancer is the most prevalent, followed by bile duct cancer, pancreatic cancer, gastric cancer, and colorectal cancer^2)^. According to the guidelines for palliative care among patients with cancer, early palliative care referral is particularly recommended at the time of diagnosis for cancers with poor prognosis, such as lung cancer without actionable driver mutations and pancreatic cancer, rather than for cancers with a longer disease trajectory or curative potential^18)^. Therefore, palliative care intervention is more likely to be implemented for cancer types that inherently require early palliative care involvement.

Various cancer rehabilitation interventions are implemented in PCUs, with physical therapy, exercise, psychoeducation, and symptom management being the most common interventions^2,5,19)^. In the present study, relaxation therapy was the most common. Previous studies have revealed that patients receiving palliative care have a variety of unmet medical needs, including psychological and emotional symptoms, physical symptoms, and spiritual concerns^3,4,20,21)^. Relaxation therapy (e.g., progressive muscle relaxation, deep breathing, and guided imagery) has been reported to be an effective treatment for these unmet needs, as well as physical symptoms in patients with cancer^22,23)^. Thus, relaxation therapy is an effective component of comprehensive interventions aimed at addressing total pain and can be widely applied in cancer rehabilitation.

Patients with cancer receiving palliative care also place a high value on maintaining independence in activities of daily living (ADL) and instrumental ADL (IADL)^24)^. Our survey revealed that walking exercises, basic mobility training, resistance exercises, and ROM exercises were among the most commonly implemented interventions, suggesting that these practices align with the needs of patients with cancer in the palliative care phase. Furthermore, support for QoL, *ikigai*, and temporary discharge from the PCU were observed as distinctive elements of palliative care, suggesting that PCUs have distinct characteristics from inpatient and outpatient cancer rehabilitation^12,13)^. *Ikigai*, a unique Japanese concept representing a sense of meaning and purpose in life, is a perspective that has not been explored in other countries. Although having *ikigai* may promote health and well-being among Japanese older adults^25)^, its impact in PCU settings has not yet been thoroughly examined. Future research should investigate the impact of *ikigai* in PCU settings and compare it with similar concepts in other countries, along with exploring an individualized approach to the diverse *ikigai* that Japanese people may hold.

Furthermore, we observed a notable disparity in SLT-specific interventions: dysphagia rehabilitation accounted for 26.0%, whereas support of cognitive function accounted for only 3.7%. Thus, SLT interventions primarily focused on addressing dysphagia, with limited attention to cognitive function and communication support. Given the importance of these aspects in maintaining and improving QoL, both interventions should be systematically developed in the future.

The most frequent intervention duration in the PCU was 20–40 min per session, similar to that in inpatient and outpatient cancer rehabilitation in Japan^12,13)^. In terms of intervention frequency, inpatient cancer rehabilitation was conducted 5–6 times per week at many facilities^12)^. In contrast, the majority of PCUs in this survey conducted rehabilitation 3–4 times per week. The lower intervention frequency in PCUs compared to that in general wards may be due to patients’ physical symptoms and overall condition, which could make frequent interventions more challenging. Additionally, the unavailability of reimbursement may have led to staffing inadequacies, which might have limited the ability to implement more frequent interventions.

The results of our survey underscore that relaxation therapy is widely used in PCUs to manage symptoms in advanced and palliative phases and that interventions to maintain ADLs and IADLs—the primary outcomes of rehabilitation, which are in high demand—are often provided. Although the present study analyzed the frequency and time of interventions, the underlying reasons could not be directly examined. Furthermore, stratified analyses according to facility characteristics did not identify clear differences in intervention frequency or duration. Challenges related to personnel allocation and reimbursement may have affected the delivery of rehabilitation services^11)^. Particularly, in the area of palliative care, increasing the intensity of interventions is often difficult, and it is assumed that ensuring the frequency and duration of interventions is important to maintain and improve outcomes.

Evidence from a multicenter, prospective, randomized controlled trial conducted in PCUs across Japan demonstrated that individualized intervention of 40 min per day, delivered through Optimal and Patient-Centered Rehab Practice, was associated with less ADL decline compared with the conventional 20-min intervention^26,27)^. Therefore, providing patient-specific, time-guaranteed interventions for cancer rehabilitation in the PCU is important. Future efforts to strengthen the evidence base for cancer rehabilitation in PCUs should focus on collecting data through prospective cohort studies and well-designed intervention trials. These efforts will help remove barriers, such as the ineligibility for medical fee reimbursement for the disease and a shortage of rehabilitation staff. These efforts will also lead to adequate interventions, which will contribute to enhancing end-of-life care and patient outcomes.

While our findings do not directly evaluate policy mechanisms, they highlight structural challenges in current practices. The study results also suggest that reforms in reimbursement systems, including the introduction of disease-specific rehabilitation fees, the establishment of staffing standards for dedicated rehabilitation personnel in PCUs, and the inclusion of evaluation in medical fees for those who have completed specialized training and workforce allocation, are important areas for future policy discussions. Strengthening these structural aspects will be essential to translate clinical evidence into practice and to optimize patient-centered palliative care.

This study has some limitations. First, only a single mail reminder was used during the data collection process. In survey methodology, multiple follow-up approaches, such as telephone calls or web-based reminders, are known to improve participation rates and reduce nonresponse bias. Considering the nationwide scope and diversity of PCUs, implementing multimodal follow-up strategies might have improved the representativeness of the sample. Future survey research in this field should consider incorporating additional follow-up procedures to minimize potential nonresponse bias. Second, although the analysis suggested no significant differences in the location of facilities, hospital type, or number of beds between responder and nonresponder facilities, the possibility of unmeasured differences could not be eliminated. Third, to gain an understanding of the current state of cancer rehabilitation in PCUs, we obtained responses from rehabilitation staff in supervisory positions. However, we could not rule out the possibility that the responses might not fully reflect actual rehabilitation practices. Fourth, although the response rate for this study was relatively high at 57.4%, facilities that do not provide rehabilitation services or lack the necessary infrastructure might have been less likely to respond. Therefore, the estimated rate of rehabilitation provision might be higher than the actual rate. Fifth, the study focused exclusively on cancer rehabilitation in PCUs in Japan, which limited the generalizability of the findings to other countries or to patients with noncancer diagnoses. Sixth, the study was conducted in only a subset of Japanese PCUs, which might not fully represent the national situation. Subsequent investigations are needed to develop more comprehensive findings. Finally, although the questionnaire was carefully developed through discussions among cancer rehabilitation experts, including physiatrists, PTs, and OTs, its validity was not formally tested before implementation.

## Conclusions

In Japanese PCUs, cancer rehabilitation primarily targeted lung, hepatobiliary and pancreatic, colorectal, gastric, and breast cancers. Interventions such as relaxation therapy, walking, basic mobility training, resistance exercises, and ROM exercises were commonly implemented. Additionally, support for QoL, *ikigai* (a Japanese concept for a sense of meaning and purpose in life), and temporary discharge from the PCU were observed as distinctive elements of palliative care. At many facilities, rehabilitation was provided for 20–40 min per session, 3–4 times per week. Intervention frequency and duration are limited, and systems that can provide adequate interventions are required.

Our findings highlight the necessity for clinical research to develop robust evidence supporting patient-centered rehabilitation in PCUs, thereby ensuring the implementation of adequate, patient-centered interventions. Such evidence can guide clinical practice and inform policy discussions on reimbursement and workforce allocation, ultimately contributing to the enhancement of palliative care for individuals with advanced cancer.

## Data Availability

The data that support the findings of this study are available from the corresponding author upon reasonable request.

## Supplementary Materials

Supplementary Material 1. A Survey on the Current State of Cancer Rehabilitation in Palliative Care Units.

Supplementary Material 2. Association between facility characteristics and cancer types.
