# Supplementary material for: Overview of Cancer Rehabilitation Provided in Palliative Care Units in Japan: A Nationwide Survey
Source: Phys Ther Res. 2026 Jun 4;29(2):125–32. doi: 10.1298/ptr.25-E10376 (PMC13624565; doi:10.1298/ptr.25-E10376)
Supplement: Supplementary file 1 [file ptr-29-125-s001.pdf]

Supplementary material 1: A Survey on the Current State of Cancer Rehabilitation in Palliative Care Units

1. Please provide an overview of your facility.

Q1. Please select the type of facility

1. University hospital
2. Cancer center
3. General hospital
4. Other

Q2. Please select the total number of beds for the facility

1. <300 beds
2. 301–600 beds
3. 601–1000beds
4. >1001beds

Q3. Please select the number of beds in the palliative care unit

1. 0 bed
2. 1–10 beds
3. 11–30 beds
4. 31–60 beds
5. >61beds

Q4. Please indicate whether your facility has a physiatrist

1. Yes
2. No

Q5. Please fill in the number of rehabilitation staffs

1. Physical therapists
2. Occupational therapists
3. Speech and language therapists

Q6. Please fill in the number of rehabilitation staffs who had completed the CAREER (Cancer Rehabilitation Educational program for Rehabilitation teams) program

1. Physical therapists
2. Occupational therapists
3. Speech and language therapists

2. Please provide details regarding the implementation of cancer rehabilitation in your facility's palliative care unit.

Q7. Do your facilities provide rehabilitation for patients with cancer in the palliative care unit?

1. Yes → Proceed to Q8
2. No → Thank you for your cooperation.

3. Please respond if your facility provides cancer rehabilitation in the palliative care unit.

Q8. Please select the rehabilitation staffs involved in the palliative care unit. (Multiple answers allowed)

1. Physical therapists
2. Occupational therapists
3. Speech and language therapists

Q9. Please select the rehabilitation system in the palliative care unit.

1. Exclusively assigned to palliative care unit
2. Working in concurrent roles
3. Both

Q10. Please select the most common types of cancer as target diseases. (Multiple answers allowed: up to top 5)

1. Brain
2. Bone and soft tissue sarcoma
3. Head and neck
4. Esophageal
5. Stomach
6. Colorectal
7. Hepatobiliary pancreatic
8. Lung
9. Hematological
10. Breast
11. Gynecological
12. Renal

Q11. Please select the most common types of intervention. (Multiple answers allowed: up to top 5)

1. Resistance exercise
2. Range of motion exercise
3. Walking exercise
4. Basic mobility training
5. ADL training
6. Pulmonary rehabilitation
7. Dysphagia rehabilitation
8. Support for lymphedema
9. Relaxation
10. Support for cognitive function
11. Support for returning home
12. Support for QoL and ikigai

Q12. Please provide the average number of cancer patients treated per day in the palliative care unit over the past month.

1. Physical therapists
2. Occupational therapists
3. Speech and language therapists

Q13. Please select the average intervention time (per session) for cancer patients in the palliative care unit over the past month.

1. <20 min
2. 20–40 min
3. >40 min

Q14. Please select the average intervention frequency (per week) for cancer patients in the palliative care unit over the past month.

1. 1-2 times/week
2. 3-4 times/week
3. 5-6 times/week
4. 7 times/week

Thank you very much for taking the time to complete our survey.
