# Supplementary material for: Overview of Cancer Rehabilitation Provided in Palliative Care Units in Japan: A Nationwide Survey
Source: Phys Ther Res. 2026 Jun 4;29(2):125–32. doi: 10.1298/ptr.25-E10376 (PMC13624565; doi:10.1298/ptr.25-E10376)
Supplement: Supplementary file 2 [file ptr-29-125-s002.pdf]

Supplementary material 2: Association between facility characteristics and cancer types

Facility type

	University hospital n=6	Cancer center n=10	General hospital n=218	Others n=4	P-value
Brain	1 (16.7%)	2 (20.0%)	32 (14.7%)	1 (25.0%)	0.911
Bone and soft tissue sarcoma	0 (0.0%)	1 (10.0%)	13 (6.0%)	1 (25.0%)	0.385
Head and neck	0 (0.0%)	3 (30.0%)	24 (11.0%)	1 (25.0%)	0.187
Esophageal	2 (33.3%)	2 (20.0%)	40 (18.3%)	0 (0.0%)	0.614
Stomach	3 (50.0%)	5 (50.0%)	150 (68.8%)	2 (50.0%)	0.401
Colorectal	4 (66.7%)	5 (50.0%)	156 (71.6%)	3 (75.0%)	0.527
Hepatobiliary pancreatic	4 (66.7%)	6 (60.0%)	173 (79.4%)	4 (100.0%)	0.293
Lung	5 (88.3%)	8 (80.0%)	183 (83.9%)	4 (100.0%)	0.829
Hematological	0 (0.0%)	1 (10.0%)	32 (14.7%)	0 (0.0%)	0.603
Breast	1 (16.7%)	7 (70.0%)	90 (41.3%)	1 (25.0%)	0.150
Gynecological	3 (50.0%)	5 (50.0%)	56 (25.7%)	1 (25.0%)	0.217
Renal	2 (33.3%)	4 (40.0%)	70 (32.1%)	2 (50.0%)	0.845

PCU beds

	1–10 beds n=10	11–30 beds n=206	31–60 beds n=18	>61beds n=1	P-value
Brain	1 (10.0%)	30 (14.6%)	3 (16.7%)	1 (100.0%)	0.113
Bone and soft tissue sarcoma	0 (0.0%)	14 (6.8%)	1 (5.6%)	0 (0.0%)	0.842
Head and neck	1 (10.0%)	27 (13.1%)	1 (5.6%)	0 (0.0%)	0.785
Esophageal	3 (30.0%)	36 (17.5%)	3 (16.7%)	0 (0.0%)	0.739
Stomach	7 (70.0%)	133 (64.6%)	14 (77.8%)	1 (100.0%)	0.596
Colorectal	8 (80.0%)	147 (71.4%)	12 (66.7%)	0 (0.0%)	0.388

Hepatobiliary pancreatic	6 (60.0%)	160 (77.7%)	16 (88.9%)	1 (100.0%)	0.333
Lung	10 (100.0%)	170 (82.5%)	17 (94.4%)	1 (100.0%)	0.269
Hematological	1 (10.0%)	30 (14.6%)	0 (0.0%)	0 (0.0%)	0.346
Breast	3 (30.0%)	85 (41.3%)	11 (61.1%)	0 (0.0%)	0.256
Gynecological	2 (20.0%)	57 (27.7%)	4 (22.2%)	0 (0.0%)	0.832
Renal	4 (40.0%)	70 (34.0%)	4 (22.2%)	1 (100.0%)	0.359

Presence of the physiatrist

	Physiatrist (yes) n=90	Physiatrist (no) n=149	P-value
Brain	17 (18.9%)	19 (12.8%)	0.199
Bone and soft tissue sarcoma	5 (5.6%)	10 (6.7%)	0.721
Head and neck	13 (14.4%)	16 (10.7%)	0.395
Esophageal	19 (21.1%)	25 (16.8%)	0.402
Stomach	59 (65.6%)	100 (67.1%)	0.805
Colorectal	69 (76.7%)	102 (68.5%)	0.173
Hepatobiliary pancreatic	67 (74.4%)	120 (80.5%)	0.269
Lung	75 (83.3%)	128 (85.9%)	0.590
Hematological	16 (17.8%)	16 (10.7%)	0.122
Breast	37 (41.1%)	61 (40.9%)	0.979
Gynecological	28 (31.1%)	36 (24.2%)	0.240
Renal	24 (26.7%)	56 (37.6%)	0.083

Rehabilitation implementation system

	Exclusively assigned to PCU n=6	Working in concurrent roles n=216	Both n=20	P-value
Brain	0 (0.0%)	33 (15.3%)	3 (15.0%)	0.584
Bone and soft tissue sarcoma	0 (0.0%)	14 (6.5%)	1 (5.0%)	0.788
Head and neck	2 (33.3%)	25 (11.6%)	2 (10.0%)	0.259
Esophageal	1 (16.7%)	41 (19.0%)	2 (10.0%)	0.606
Stomach	2 (33.3%)	146 (67.6%)	13 (65.0%)	0.212
Colorectal	5 (83.3%)	152 (70.4%)	15 (75.0%)	0.726
Hepatobiliary pancreatic	6 (100.0%)	166 (76.9%)	18 (90.0%)	0.169
Lung	5 (83.3%)	180 (83.3%)	19 (95.0%)	0.389
Hematological	0 (0.0%)	31 (14.4%)	2 (10.0%)	0.531
Breast	3 (50.0%)	89 (41.2%)	9 (45.0%)	0.869
Gynecological	2 (33.3%)	60 (27.8%)	3 (15.0%)	0.438
Renal	4 (66.7%)	69 (31.9%)	7 (35.0%)	0.200

Association between facility characteristics and intervention type

Facility type

	University hospital n=6	Cancer center n=10	General hospital n=218	Others n=4	P-value
Resistance exercise	3 (50.0%)	7 (70.0%)	116 (53.2%)	1 (25.0%)	0.487
ROM exercise	4 (66.7%)	5 (50.0%)	109 (50.0%)	1 (25.0%)	0.644
Walking exercise	3 (50.0%)	8 (80.0%)	144 (66.1%)	3 (75.0%)	0.638
Basic mobility training	5 (83.3%)	7 (70.0%)	119 (54.6%)	1 (25.0%)	0.230
ADL training	3 (50.0%)	6 (60.0%)	98 (45.0%)	1 (25.0%)	0.659
Pulmonary rehabilitation	2 (33.3%)	2 (20.0%)	49 (22.5%)	0 (0.0%)	0.658
Dysphagia rehabilitation	0 (0.0%)	3 (30.0%)	60 (27.5%)	0 (0.0%)	0.285
Support for lymphedema	0 (0.0%)	0 (0.0%)	47 (21.6%)	2 (50.0%)	0.094
Relaxation	5 (83.3%)	4 (40.0%)	152 (69.7%)	4 (100.0%)	0.094
Support for cognitive function	0 (0.0%)	0 (0.0%)	9 (4.1%)	0 (0.0%)	0.836
Support for returning home	2 (33.3%)	3 (30.0%)	78 (35.8%)	2 (50.0%)	0.916
Support for QoL and ikigai	1 (16.7%)	3 (30.0%)	78 (35.8%)	3 (75.0%)	0.286

PCU beds

	1–10 beds n=10	11–30 beds n=206	31–60 beds n=18	>61beds n=1	P-value
Resistance exercise	5 (50.0%)	105 (51.0%)	12 (66.7%)	0 (0.0%)	0.434
ROM exercise	7 (70.0%)	101 (49.0%)	11 (61.1%)	0 (0.0%)	0.317
Walking exercise	5 (50.0%)	135 (65.5%)	14 (77.8%)	1 (100.0%)	0.426
Basic mobility training	7 (70.0%)	109 (52.9%)	12 (66.7%)	0 (0.0%)	0.327
ADL training	5 (50.0%)	91 (44.2%)	9 (50.0%)	0 (0.0%)	0.765
Pulmonary rehabilitation	2 (20.0%)	49 (23.8%)	3 (16.7%)	0 (0.0%)	0.842

Dysphagia rehabilitation	3 (30.0%)	54 (26.2%)	6 (33.3%)	0 (0.0%)	0.838
Support for lymphedema	1 (10.0%)	45 (21.8%)	1 (5.6%)	1 (100.0%)	0.064
Relaxation	8 (80.0%)[#]	150 (72.8%)[#]	7 (38.9%)	1 (100.0%)[#]	0.018
Support for cognitive function	0 (0.0%)	9 (4.4%)	0 (0.0%)	0 (0.0%)	0.725
Support for returning home	3 (30.0%)	74 (35.9%)	5 (27.8%)	1 (100.0%)	0.487
Support for QoL and ikigai	3 (30.0%)	74 (35.9%)	6 (33.3%)	1 (100.0%)	0.575

Presence of the physiatrist

	Physiatrist (yes) n=90	Physiatrist (no) n=149	P-value
Resistance exercise	42 (46.7%)	86 (57.7%)	0.097
ROM exercise	48 (53.3%)	74 (49.7%)	0.582
Walking exercise	62 (68.9%)	95 (63.8%)	0.418
Basic mobility training	47 (52.2%)	85 (57.0%)	0.467
ADL training	36 (40.0%)	70 (47.0%)	0.293
Pulmonary rehabilitation	26 (28.9%)	29 (19.5%)	0.093
Dysphagia rehabilitation	21 (23.3%)	41 (27.5%)	0.475
Support for lymphedema	23 (25.6%)	26 (17.4%)	0.133
Relaxation	64 (71.1%)	102 (68.5%)	0.666
Support for cognitive function	4 (4.4%)	4 (2.7%)	0.464
Support for returning home	31 (34.4%)	53 (35.6%)	0.860
Support for QoL and ikigai	35 (38.9%)	50 (33.6%)	0.404

Rehabilitation implementation system

	Exclusively assigned to PCU n=6	Working in concurrent roles n=216	Both n=20	P-value

Resistance exercise	3 (50.0%)	118 (54.6%)	7 (35.0%)	0.240
ROM exercise	2 (33.3%)	111 (51.4%)	9 (45.0%)	0.601
Walking exercise	2 (33.3%)	146 (67.6%)	11 (55.0%)	0.126
Basic mobility training	1 (16.7%)	126 (58.3%)	7 (35.0%)	0.021
ADL training	1 (16.7%)	101 (46.8%)	7 (35.0%)	0.221
Pulmonary rehabilitation	0 (0.0%)	50 (23.1%)	5 (25.0%)	0.397
Dysphagia rehabilitation	0 (0.0%)	57 (26.4%)	6 (30.0%)	0.318
Support for lymphedema	3 (50.0%)	41 (19.0%)	6 (30.0%)	0.101
Relaxation	6 (100.0%)	149 (69.0%)	14 (70.0%)	0.264
Support for cognitive function	1 (16.7%)	7 (3.2%)	1 (5.0%)	0.219
Support for returning home	4 (66.7%)	73 (33.8%)	9 (45.0%)	0.165
Support for QoL and ikigai	1 (16.7%)	72 (33.3%)[#]	13 (65.0%)[#]	0.011

Association between facility characteristics and intervention time

Facility type

	University hospital n=5	Cancer center n=10	General hospital n=216	Others n=4	P-value
<20 min	1 (20.0%)	4 (40.0%)	49 (22.7%)	2 (50.0%)	0.722
20–40 min	4 (80.0%)	6 (60.0%)	159 (73.6%)	2 (50.0%)	
>40 min	0 (0.0%)	0 (0.0%)	8 (3.7%)	0 (0.0%)	

PCU beds

	1–10 beds n=10	11–30 beds n=203	31–60 beds n=18	>61beds n=1	P-value
<20 min	3 (30.0%)	48 (23.6%)	6 (33.3%)	0 (0.0%)	0.916
20–40 min	7 (70.0%)	148 (72.9%)	11 (61.1%)	1 (100.0%)	
>40 min	0 (0.0%)	7 (3.4%)	1 (5.6%)	0 (0.0%)	

Presence of the physiatrist

	Physiatrist (yes) n=89	Physiatrist (no) n=148	P-value
<20 min	16 (18.0%)	41 (27.7%)	0.202
20–40 min	69 (77.5%)	103 (69.6%)	
>40 min	4 (4.5%)	4 (2.7%)	

Rehabilitation implementation system

	Exclusively assigned to PCU n=6	Working in concurrent roles n=213	Both n=20	P-value
<20 min	3 (50.0%)	51 (23.9%)	3 (15.0%)	0.119

20–40 min	2 (33.3%)	156 (73.2%)	16 (80.0%)	
>40 min	1 (16.7%)	6 (2.8%)	1 (5.0%)	

Association between facility characteristics and intervention frequency

Facility type

	University hospital<br>n=5	Cancer center<br>n=10	General hospital<br>n=217	Others<br>n=4	P-value
1-2 times/week	2 (40.0%)	2 (20.0%)	44 (20.3%)	0 (0.0%)	0.149
3-4 times/week	2 (40.0%)	6 (60.0%)	106 (48.8%)	4 (100.0%)	
5-6 times/week	0 (0.0%)	2 (20.0%)	62 (28.6%)	0 (0.0%)	
7 times/week	1 (20.0%)	0 (0.0%)	5 (2.3%)	0 (0.0%)	

PCU beds

	1–10 beds<br>n=9	11–30 beds<br>n=205	31–60 beds<br>n=18	>61beds<br>n=1	P-value
1-2 times/week	1 (11.1%)	46 (22.4%)	3 (16.7%)	0 (0.0%)	0.464
3-4 times/week	4 (44.4%)	100 (48.8%)	12 (66.7%)	1 (100.0%)	
5-6 times/week	3 (33.3%)	56 (27.3%)	3 (16.7%)	0 (0.0%)	
7 times/week	1 (11.1%)	3 (1.5%)	0 (0.0%)	0 (0.0%)	

Presence of the physiatrist

	Physiatrist (yes)<br>n=90	Physiatrist (no)<br>n=147	P-value
1-2 times/week	17 (18.9%)	33 (22.4%)	0.438
3-4 times/week	50 (55.6%)	68 (46.3%)	
5-6 times/week	20 (22.2%)	43 (29.3%)	
7 times/week	3 (3.3%)	3 (2.0%)	

Rehabilitation implementation system

	Exclusively assigned to PCU n=6	Working in concurrent roles n=214	Both n=20	P-value
1-2 times/week	1 (16.7%)	48 (22.4%)	2 (10.0%)	0.194
3-4 times/week	2 (33.3%)	107 (50.0%)	10 (50.0%)	
5-6 times/week	2 (33.3%)	54 (25.2%)	8 (40.0%)	
7 times/week	1 (16.7%)	5 (2.3%)	0 (0.0%)	
